# UPLC-MS/MS Analytical Method for the Simultaneous Quantification of Diazepam, Nordazepam, and Oxazepam in Patients With Alcohol Dependence

**DOI:** 10.31083/AP38973

**Published:** 2026-02-26

**Authors:** Xiao-Lin Li, Wan-Ting Huang, Xiao-Jia Ni, Hao-Yang Lu, Shan-Qing Huang, Yu-Qing Li, Huan-Shan Xie, Yu-Guan Wen, Zhan-Zhang Wang, De-Wei Shang

**Affiliations:** ^1^Department of Pharmacy, The Affiliated Brain Hospital, Guangzhou Medical University, 510370 Guangzhou, Guangdong, China; ^2^Department of Pharmacy, The Third People’s Hospital of Zhuhai, 519000 Zhuhai, Guangdong, China; ^3^Key Laboratory of Neurogenetics and Channelopathies of Guangdong Province and the Ministry of Education of China, Guangzhou Medical University, 510370 Guangzhou, Guangdong, China

**Keywords:** diazepam, nordazepam, oxazepam, ultra-performance liquid chromatography–mass spectrometry, alcohol dependence

## Abstract

**Background:**

To establish a method for the simultaneous quantification of diazepam (DIA) and its active metabolites, nordazepam (NorD) and oxazepam (OXAZ), and provide a reference range for therapeutic concentrations in patients with alcohol dependence.

**Methods:**

Simple and direct protein precipitation was used to extract the biological samples. Subsequent separation was performed on an Agilent XDB-C18 column (50 mm × 4.6 mm, 1.8 μm) with a column temperature maintained at 35 °C and a flow rate of 0.5 mL/min via ultra-high performance liquid chromatography–tandem mass spectrometry (UPLC-MS/MS). The mobile phase consisted of methanol–water containing 5 mM ammonium formate (75:25, v/v). Detection was conducted using electrospray ionization in multiple reaction monitoring modes: *m/z* 284.6→193.2 for DIA, *m/z* 270.5→140.1 for NorD, *m/z* 286.9→241.1 for OXAZ, *m/z* 289.6→198.2 for DIA-D5, *m/z *275.5→140.0 for NorD-D5, and *m/z* 291.9→246.1 for OXAZ-D5. The linear response range for DIA, NorD, and OXAZ was 1–1500 ng/mL.

**Results:**

The key parameters of the bioanalytical method were validated: the average extraction recovery was 95%–101% (CV <6%); calibration curves exhibited good linearity over the concentration range (R^2^ ≥0.99 for all analytes); accuracy was within 85%–115%; and intra-day and inter-day precision were satisfactory (CVs <15%). The concentrations of analytes in 26 routine therapeutic drug monitoring (TDM) samples from patients with alcohol dependence were determined.

**Conclusions:**

We developed and validated a rapid, simple, and economic UPLC-MS/MS method for the quantification of DIA, NorD, and OXAZ in human serum. The method is well-suited for the determination of serum levels of DIA and its active metabolites in patients with alcohol dependence, and could be further applied to TDM and subsequent studies.

## Main Points

1. Exploring and applying the therapeutic concentration reference range of benzodiazepines for the treatment of alcohol dependence is beneficial.

2. The developed method is suitable for quantifying the serum levels of diazepam and its active metabolites in patients with alcohol dependence. Based on real-world sample data, the concentration range of the standard curve meets clinical requirements.

3. Employing one-step protein precipitation with acetonitrile for serum sample preparation reduces solvent consumption and time, enabling rapid high-throughput quantitative analysis, which aligns with the principles of green analytical chemistry.

4. The use of deuterated internal standards enhances the specificity of analytical results and the reliability of the method.

## 1. Introduction

Alcohol dependence is the third-leading cause of death in the USA, accounting for 145,000 deaths annually [1]. Alcohol withdrawal syndrome is a life-threatening condition occurring after intentional or unintentional abrupt cessation of heavy/constant drinking [2], and is the most common reason for hospital admission in people with unhealthy alcohol use.

Benzodiazepines have one or more 6-carbon benzene rings, a 7-carbon diazepine ring, and various substituents, and are the standard treatment for alcohol withdrawal syndromes [3]. Benzodiazepines bind to benzodiazepine receptors on the γ-aminobutyric acid (GABA) receptor complex and modulate the central nervous hyperactivity by interacting with the GABA system [3]. Diazepam (7-chloro-1,3-dihydro-1-methyl-5-phenyl-2H-1,4-benzodiazepin-2-one; DIA) is the preferred benzodiazepine for treatment of alcohol withdrawal syndrome. DIA exerts its effects via stimulation of GABA and glutamate receptors, supported by Level A evidence [4]. It is frequently used as an adjunctive treatment for several psychiatric illnesses including seizures, alcohol withdrawal, and anxiety [5], and is listed as a core drug on the essential medicines list for managing anxiety disorders [6] by the World Health Organization. Oxazepam (7-chloro-2,3-dihydro-2-oxo-5-phenyl-1H-1,4-benzodiazepin-3-ol; OXAZ) is a benzodiazepine hypnotic and sedative with antiepileptic, anxiolytic, and sedative–hypnotic properties, which acts on the GABA and its receptors (GABA _A_) and enhances the activity of the GABA system.

DIA is metabolized by CYP3A and CYP2C19 enzymes into a major active metabolite, nordazepam (N-desmethyldiazepam; NorD), and a minor active metabolite, temazepam. NorD is further metabolized to another active metabolite, OXAZ. Temazepam and OXAZ are ultimately converted to glucuronide conjugates [7]. However, the two most active species in serum are still DIA and NorD [8]. This can be partly attributed to their long elimination half-lives: 20–50 h for DIA and 30–200 h for NorD, enabling their serum levels to remain detectable days after the previous dose [9]. Because the metabolites of DIA are all physiologically active, the determination of serum concentrations of DIA alone cannot fully and accurately predict drug effects. DIA and OXAZ have similar pharmacological mechanisms, and are often used as alternative drugs and sometimes in combination or sequential therapy, to achieve greater efficacy and better remission of alcohol dependence, which raises additional concerns about drug–drug interactions. In addition, the body’s sensitivity to these analytes may decrease due to cross-resistance, potentially leading to reduced drug efficacy when concentrations fall below the therapeutic threshold. If the dose of the drug is increased, the benefits should be weighed against the risks of toxic side effects. Furthermore, for patients with alcohol dependence, long-term drinking leads to changes in metabolic function that may affect the metabolism of endogenous substances and drugs. Metabolic dysfunction may lead to drug accumulation. Therefore, to reach the therapeutic concentration and avoid adverse reactions caused by drug accumulation and interaction, it is necessary to measure the concentration of DIA and its active metabolites simultaneously [10], as suggested by the Arbeitsgemeinschaft für Neuropsychopharmakologie und Pharmakopsychiatri (AGNP) guidelines. However, the reference range of therapeutic concentrations of benzodiazepines for the treatment of alcohol dependence has not been established. Therefore, there is a need to develop a simple and reliable method for the simultaneous quantification of DIA and its active metabolites, and for the development of therapeutic reference ranges of these analytes in the clinical management of alcohol dependence.

The concentrations of DIA and its active metabolites in human blood have been determined by several analytical techniques, such as high-performance liquid chromatography (HPLC) [11, 12], ultra-HPLC (UPLC) [13], or gas chromatography coupled with a ultraviolet (UV) detector or mass spectrometry (MS) [14, 15, 16, 17, 18, 19, 20]. For sample preparation, liquid–liquid extraction (LLE) [11], solid–phase extraction (SPE) [12], or protein precipitation (PP) [13] was used. Most of the recently published methods used time-consuming LLE [11, 21, 22, 23, 24] or SPE [12, 14, 18, 20, 25] to extract analytes and achieved high-throughput screening of multiple drugs in human blood. A recent method by Barone *et al*. [13] used PP to extract 68 analytes from postmortem blood. Tok *et al*. [26] concluded that the PP was environmentally friendly and sustainable by conducting AGREEPrep scoring of different sample preparation steps. Qandeel *et al*. [27] developed an eco-friendly proton nuclear magnetic resonance method to quantify DIA in tablets, with a linearity range of 0.25–15 mg/mL. However, the linearity ranges of most of these methods are not suitable for measurement of therapeutic concentration in people with alcohol dependence [11, 12, 13, 14, 22, 27, 28]. This is because the reported range of DIA is 200–1500 ng/mL [9, 28, 29] and the ratio of DIA to NorD ranges from 0.028 to 2.80 in postmortem patients [30]. Besides, some methods lacked deuterated internal standards (ISs) [13, 14], required a large sample volume (≥100 µL) [11, 12, 18, 22, 23, 25, 28], or lacked metabolite detection [14]. **Supplementary Table 1** summarizes and contrasts the analytical methods used in different studies. Overall, we need to optimize the assay protocol to create a simple, rapid, and economical bioanalytical method to quantify DIA, NorD, and OXAZ, which meet the requirement of clinical examination.

The purpose of this study was to develop and validate a rapid, simple, and economic method to simultaneously determine DIA and its metabolites in human serum using UPLC-tandem MS (UPLC-MS/MS). The method was used for serum drug concentration monitoring in patients with alcohol dependence treated with DIA or OXAZ. Our results have important implications for the individualized treatment of alcohol dependence.

## 2. Materials and Methods

### 2.1 Chemicals and Reagents

DIA (lot: FE12021903, purity: 99.9%), NorD (lot: FE10012008, purity: ≥99.25%), OXAZ (lot: FE07022005, purity: 99.57%), DIA-D5 sodium (IS, lot: FE06292011, purity: 99.19%), NorD-D5 sodium (IS, lot: FE09102002, purity: ≥98.44%), and OXAZ-D5 sodium (IS, lot: FE03312003, purity: 99.17%) were purchased from Guangzhou Belt Scientific Equipment Co., Ltd. (Guangdong, China); a distributor for Cerilliant Corporation. Sigma–Aldrich LLC (St. Louis, MO, USA) provided acetonitrile, methanol, and ammonium formate of HPLC grade. Deionized water was produced using a Milli-Q academic reagent-grade water purification system (Millipore Corporation, Billerica, MA, USA).

### 2.2 Instrumentation

Chromatography using the Shimadzu 30A HPLC system (Shimadzu Corporation, Kyoto, Japan) was performed using two LC-30AD pumps, SIL-30ACMP autosampler, SPD-M30A detector, and CTO-30A column oven. With a flow rate of 0.5 mL/min, the mobile phase was 75% methanol (25/75, V/V, water/methanol) containing 5 mM ammonium formate. Analytes were separated on an Agilent XDB-C18 (50 mm × 4.6 mm, 1.8 µm, Agilent Technologies Inc., Santa Clara, CA, USA) analytical column kept at 35 °C. The LC ran for 2.8 min with an injection volume of 5 µL.

**Fig. 1.**
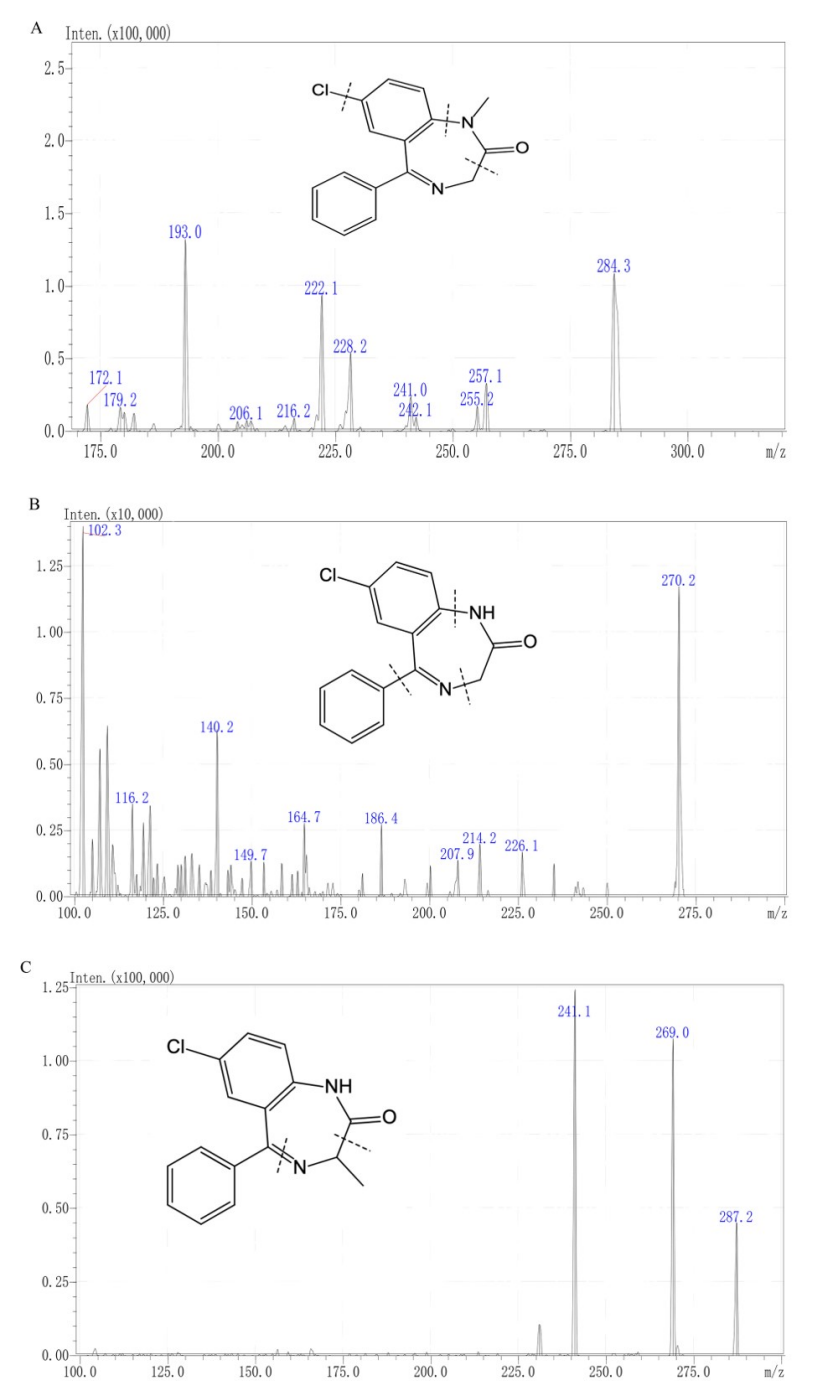
**Chemical structures and fragmentation sites of (A) diazepam, (B) nordazepam, and (C) oxazepam**.

MS was performed on an MS-8050 triple quadrupole mass spectrometer (Shimadzu). In the precursor scan mode, the mass spectrometer used [M+H]^+^ as the precursor ion because it provided the strongest signal reaction. For quantification, based on electrospray ionization (ESI), the triple quadrupole tandem mass spectrometer performed quantitative analysis in multiple reaction monitoring (MRM) mode. The optimized transitions for the product ions that were to be scanned at a different collision energy were *m/z*284.6⟶193.2 for DIA, *m/z* 289.6⟶198.2 for DIA-D5, *m/z* 270.5⟶140.1 for NorD, and *m/z* 275.5⟶140.0 for NorD-D5, *m/z* 286.9⟶241.1 for OXAZ, and *m/z* 291.9⟶246.1 for OXAZ-D5 (Fig. 1). MS conditions were optimized as follows: desolvation line temperature 250 °C; heat block temperature 400 °C; conversion dynode voltage 6 kV; interface voltage 4.5 kV; nebulizing gas (nitrogen) 3 L/min; collision gas (argon) 230 kPa; and drying gas (nitrogen) 10 L/min.

### 2.3 Preparation of Stock Solutions, Calibration Standards, and Quality Control Samples

DIA (1 mg/mL), NorD (1 mg/mL), and OXAZ (1 mg/mL) stock solutions were prepared separately using 50% methanol and stored at 4 °C. To achieve appropriate concentrations, standard curve working solutions and quality control (QC) working solutions of three analytes were created by serially diluting stock solutions with 50% methanol aqueous solution. The working solution concentrations of three analytes were 20, 40, 300, 2000, 10,000, 15,000, 25,000, and 30,000 ng/mL. Four concentration levels, 60, 1000, 5000, and 20,000 ng/mL, were selected as the QC for serum samples. DIA-D5, NorD-D5 and OXAZ-D5 were dissolved in pure methanol to obtain deuterated IS stock solutions, all at a concentration of 1 mg/mL. Through a series of dilutions, the mixed IS working solutions were finally obtained (DIA-D5 200 ng/mL, NorD-D5 5000 ng/mL, and OXAZ-D5 200 ng/mL).

Calibration and QC samples were prepared by mixing 95 µL blank serum with 5 µL working solutions in a 2-mL Eppendorf tube, yielding final concentrations of 1, 2, 15, 100, 500, 750, 1250, and 1500 ng/mL for the calibration samples, and 3 ng/mL (low QC, LQC), 50 ng/mL (general medium QC, GMQC), 250 ng/mL (medium QC, MQC), and 1000 ng/mL (high QC, HQC) were obtained for the QC samples.

### 2.4 Sample Preparation

Protein precipitation was used for serum pretreatment. One hundred microliters of each sample was added to 20 µL of the mixed IS working solution and vortexed (XW-80A, Shanghai Medical University Instrument Manufacturer, Shanghai, China) for 15 s. Subsequently, 500 µL of acetonitrile was added, and the samples were vortexed again for 15 s to perform deproteinization. The prepared mixture was centrifuged at 20,238 ×g for 5 min using a Centrifuge 5424 (Eppendorf AG, Hamburg, Germany) before a sample of the supernatant (5 µL) was injected into the UPLC-MS/MS apparatus.

### 2.5 Method Validation

The UPLC-MS/MS approach followed the Pharmacopoeia of the People’s Republic of China (2020 Edition) of the National Medical Products Administration and the principles of Guidance for Industry Bioanalytical Method Validation by the United States Food and Drug Administration.

#### 2.5.1 Selectivity and Sensitivity

Six batches of the MRM chromatograms of unmixed, drug-free serum samples collected from various individuals were compared to evaluate the selectivity of the method as follows: (1) human serum without either analyte or IS; (2) blank serum spiked with mixed IS; (3) the upper limit of quantification (ULOQ) of each analyte spiked without the mixed IS; (4) the lower limit of quantification (LLOQ) of each analyte spiked with the mixed IS; and (5) the real patient serum samples. The peak area of the interfering component was considered acceptable when its response was <5% of the peak area of the IS and 20% of the peak area corresponding to the LLOQ of the analyte. Sensitivity was defined as the lowest measurable serum concentration (LLOQ), which had an acceptable signal-to-noise ratio (≥10 times the baseline noise level), precision (expressed relative standard deviation, RSD ≤20%), and accuracy (within ±20%).

#### 2.5.2 Linearity

Linearity was assessed by analyzing calibration curves of human serum from replicates of three separate runs. During the method validation and application process, a new calibration curve needed to be created for each quantitative batch. Each calibration curve was accompanied by a blank matrix sample without any analyte or IS, which was referred to as a double blank sample, and an IS-treated blank matrix sample to demonstrate the absence of interference from analytes and the IS. The linearity evaluation of the calibration curve, however, should not include the above blank samples. The linear weighted model was adopted with a weighting factor of 1/χ^2^. A satisfactory correlation was typically indicated by a determination coefficient (R^2^) >0.99. For the back-calculated concentrations of the calibrators, except those at the LLOQ, which should be within ±20% of the nominal value, the others should be within ±15% of the nominal value.

#### 2.5.3 Accuracy and Precision

Three replicate experiments over 2 days were used to assess the between- and within-batch precision and accuracy at five distinct concentration levels (LQC, GMQC, MQC, HQC, and LLOQ). At least five samples were processed for each concentration level. A fresh calibration curve was used to determine the concentrations of samples in each batch. The coefficient of variation (CV) was calculated to assess both intra- and inter-batch precisions. For QC samples, the precision was considered acceptable when the CV was within 15%. In the case of LLOQ, a CV within 20% was regarded acceptable. Regarding the intra- and inter-batch accuracies, for the QC samples, values within the range of 85%–115% were considered acceptable. For LLOQ, the acceptable accuracy range was 80%–120%.

#### 2.5.4 Matrix Effect and Recovery 

To assess the impact of the matrix on the assay outcomes at two concentration levels, six batches of blank serum from various sources were used (LQC and HQC). The response of samples where IS and analyte solution were added to the drug-free biological matrix extracted by organic solvent (post-spiked samples) was compared to that of analyte and IS solution added to water (untreated samples). Considering the diverse patient scenarios in practice, we also investigated matrix effects in the hemolyzed serum sample and hyperlipidemic sample. The lipid emulsion was mixed with drug-free human serum to collect hyperlipidemic serum with an ultimate fat level of 0.6%. Erythrocyte sedimentation was added to pooled blank human serum to generate hemolyzed serum containing 3% red blood cells. Each batch of blank serum had its peak area ratio determined, and the RSD was limited to 15%. The peak area ratios of post-spiked samples at two concentrations (LQC and HQC) to untreated samples were compared to determine the matrix effect, which was measured as a percentage [matrix factor, MF (%) = A_Post-spiked_/A_Untreated_ × 100%]. The matrix effect was quantified using the IS normalized matrix factor, which was the ratio of the matrix effects of the analyte and the IS [IS normalized matrix factor (%) = MF_Analyte_/MF_IS_ × 100%]. The IS-normalized CV of the matrix factor, which was derived from six batches, should fall within 15% of the nominal concentration. Peak areas from the QC samples at two concentrations and those from post-spiked samples were compared to evaluate the extraction recovery. To fulfill the need of clinical practice, the matrix effect of the other samples should be considered, such as hyperlipidemic and hemolyzed serum.

#### 2.5.5 Stability 

A newly prepared calibration curve was utilized to calculate the concentrations of QC samples of three analytes under the specified storage conditions. Three QC samples were prepared for each QC level. The stability of the analyte was considered satisfactory if the calculated concentration did not differ from the corresponding reference sample concentration. Specifically, the requirement was that the deviation of the mean value of each concentration from the nominal concentration should be within ±15%.

Short-term stability was evaluated by storing the QC samples (LQC and HQC) at room temperature for 24 h, as well as subjecting them to three freeze-thaw cycles at –80 °C. The stability of the autosampler was determined by placing the treated QC sample in the autosampler for ~24 h. Regarding long-term stability, the QC samples (LQC and HQC) were tested after 7 and 24 days of storage at –80 °C.

### 2.6 Serum Concentrations in Patients With Alcohol Dependence

The present approach was used to conduct routine therapeutic drug monitoring (TDM) in 26 inpatients with alcohol dependence. The requirement for written informed consent was waived by the Ethics Committee. All patients received DIA and/or OXAZ during the concentration monitoring period, and serum samples were collected using red-topped tubes without ethylenediaminetetraacetic acid (EDTA). All samples were trough samples collected before the next dosing, confirming that the patients’ serum drug concentrations had reached steady-state levels. All samples were preprocessed before analysis. The processing procedure was as follows: the serum samples were centrifuged at 1760 ×g for 3 min (Centrifuge 5424, Eppendorf AG, Germany), and the upper layer of serum was aspirated into a centrifuge tube and stored at –80 °C. The samples were processed in accordance with the procedure described in Section 2.4, and the serum concentration of the samples was determined using a freshly prepared standard curve.

## 3. Results

### 3.1 Method Development 

The isotopes of the target drugs, namely DIA-D5, NorD-D5, and OXAZ-D5, were used as ISs. Compared with other ISs, the deuterated ISs were selected to minimize the fluctuating influence of factors such as injection volume, analytical conditions, instrument response, and interference from endogenous substances in the sample. In the methodological exploration stage, NorD-D5 had a low response, and DIA-D5 and OXAZ-D5 had high response. When the appropriate IS concentration was selected, the concentration of NorD-D5 was increased to control the signal contribution of the IS to the analyte to not surpass 20% of the LLOQ response and not exceed 5% of the response of the IS. This ensured the accuracy of the quantitative analysis.

A mixed solution of DIA, NorD, and OXAZ was directly injected into the system to compare the reactions of the three target analytes to various mobile phases. A C18 column separated the analytes from the serum matrix. During development, methanol–water and acetonitrile–water elution procedures were used to optimize the separation of DIA, NorD, and OXAZ. Compared to acetonitrile, methanol dramatically increased the response to DIA, NorD, and OXAZ. When the amount of methanol in the mobile phase was large, the analyte response was high, and the peak shape was acceptable. When the methanol ratio was reduced, the analyte response decreased, the peak of OXAZ was distorted, and the separation effect was poor. Therefore, in this investigation, the elution system was methanol–water. OXAZ was significantly affected by the matrix components when methanol–water (85:15, v/v) was used for elution. When the proportion of the water was increased to 20%, OXAZ was able to separate from the interfering substances. Ultimately, the separation effect of OXAZ, NorD, and DIA met the detection criteria using a methanol–water system with a 25% water phase ratio. Most reported techniques typically include proton donors such as ammonium formate or formic acid [12, 13]. The analyte response was improved by adding ammonium formate to 75% methanol. We also added formic acid to the elution process to increase the drug response. For example: (1) 75% methanol (containing 0.1% formic acid) (solvent A)-methanol (solvent B); (2) 75% methanol (5 mmol/L ammonium formate) (solvent A)-methanol(solvent B); (3) 75% methanol (containing 0.1% formic acid, 5 mmol/L ammonium formate) (solvent A)-methanol(solvent B); and (4) 75% methanol (containing 0.1% formic acid, 5 mmol/L ammonium formate) (solvent A)-methanol (containing 0.1% formic acid) (solvent B). Comparison of the response of different analytes to mobile phase composition and elution gradients showed that using only one mobile phase, namely methanol–water solution (75:25, v/v) containing 5 mmol/L ammonium formate, a high response, and good chromatographic peaks were obtained in the isocratic elution process. Considering the large volume of daily TDM samples, simple and fast quantification methods were needed. The preparation processes of SPE and LLE are time-consuming and uneconomical compared to PP. Therefore, in this study, serum samples were prepared by acetonitrile precipitation with an acetonitrile-to-serum ratio of 5:1.

### 3.2 Concentration Range of the Standard Curve Design

The estimated range of the standard curve was determined according to the response of the instrument to the detection of target compounds, the sensitivity and stability of the method, the serum concentration data from the literature [9, 30, 31], and the actual concentration of the measured samples. During methodology exploration, to determine the lowest concentration, mixed samples of three target compounds at a concentration of 1, 2, and 5 ng/mL were tested first. DIA, NorD, and OXAZ could be detected at 1 ng/mL; the sensitivity of MS was sufficient; and the response was acceptable. The metabolism of DIA exhibited significant individual variation, with the metabolic concentration varying by up to 30-fold. Based on the actual concentrations determined in clinical samples, the DIA concentrations were close to 500 ng/mL for samples from patients taking DIA and within 1000 ng/mL for patients taking OXAZ. According to the AGNP guidelines [32], the therapeutic concentration was defined as 100–2500 ng/mL. The range is suitable for the treatment of anxiety and sleep disorders, which includes the concentration of active metabolites. Therefore, based on the concentration detection of some actual biological samples, the quantitative upper limit of the analysis was finally determined to be 1500 ng/mL.

### 3.3 Specificity and Selectivity

**Fig. 2.**
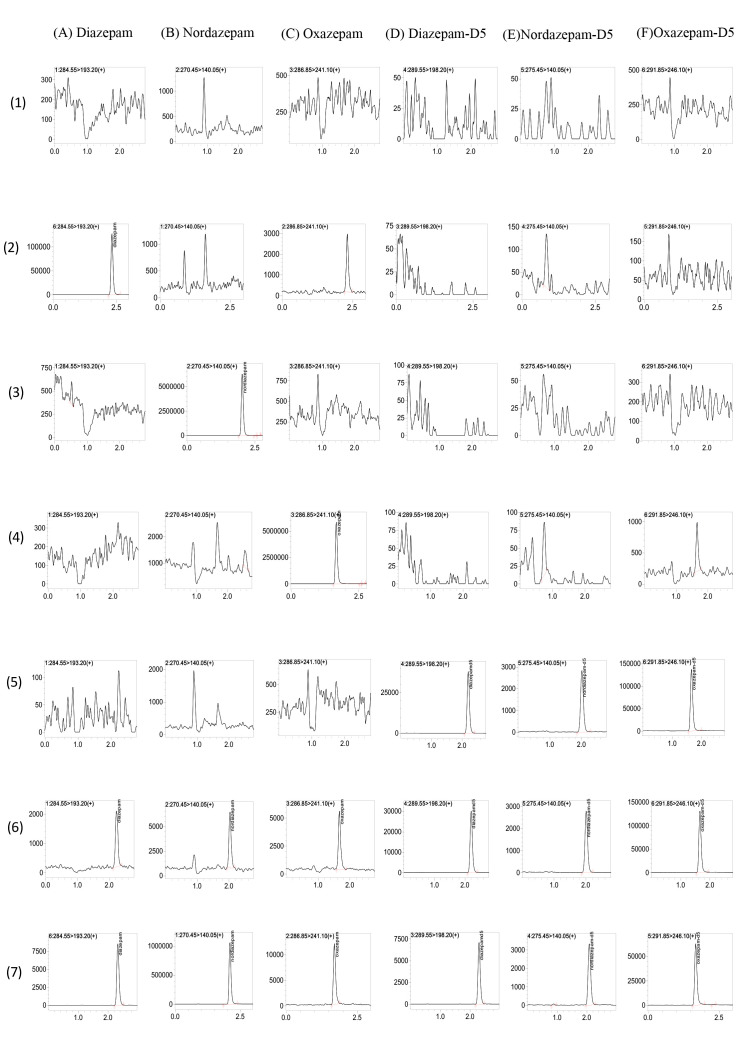
**Multiple Reaction Monitoring Chromatograms of Diazepam (DIA), Nordazepam (NorD), Oxazepam (OXAZ), Diazepam-D5, Nordazepam-D5, Oxazepam-D5**. (A–F) correspond to the six analytes/ISs; (1–7) represent sample types: (1) blank human serum, (2) blank serum spiked with DIA (upper limit of quantification; ULOQ), (3) blank serum spiked with NorD (ULOQ), (4) blank serum spiked with OXAZ (ULOQ), (5) blank serum spiked with mixed ISs, (6) blank serum spiked with mixed ISs and all analytes (lower limit of quantification, LLOQ), (7) sample serum from patient.

The chromatograms of different samples for DIA, NorD, and OXAZ are shown in Fig. 2. The retention times of DIA, NorD, and OXAZ were comparable to those of the ISs and were approximately 2.2, 2.0, and 1.6 min, respectively (Fig. 2(6)). At the relevant retention times, there was no evidence of any interference from drug-free human serum components or other test medications. An interfering peak emerged on the MRM chromatography of OXAZ in blank serum spiked with only DIA (Fig. 2(2)). The retention times of interfering peak were 2.3–2.5 min, while the real retention times of OXAZ were 1.6–1.8 min. This indicated that the interference for the detection of OXAZ was negligible.

### 3.4 Linearity and LLOQ 

The linear range of all analytes was mainly based on the guidelines [32] and the tested serum concentration in actual samples, ranging from 1 to 1500 ng/mL. The bias in the calibration accuracies for the eight concentrations of the calibration curves was within the range of ±15%. Regression analysis showed that the weight factor of all calibration curves was 1/χ^2^, and the determination coefficient (R^2^) was >0.99. After injecting a sample with a high concentration (1500 ng/mL), no significant residues were detected in the blank samples. The representative curves of DIA, NorD, and OXAZ were Y = 0.436868X + 0.00842737 (R^2^ = 0.994), Y = 280.104X + 0.823104 (R^2^ = 0.995), and Y = 0.275492X + 0.00816848 (R^2^ = 0.994), respectively.

### 3.5 Accuracy and Precision

QC samples of three analytes at five concentration levels (LLOQ, LQC, GMQC, MQC, and HQC) were evaluated in terms of accuracy and precision. For the three target compounds, the accuracy and precision of all concentration levels were within the acceptable range. Both intra- and inter-batch precisions were satisfactory, with CVs <15%. Additionally, intra- and inter-batch accuracies all met the criterion of 85–115%. The results are presented in Table 1.

**Table 1.** **Intra- and inter-batch precision and accuracy of diazepam, nordazepam and oxazepam in human serum**.

Analyte	Nominal Conc. (ng/mL)	Measured Conc. (ng/mL)	Intra-day		Measured Conc. (ng/mL)	Inter-day	
			Accuracy (%)	Precision (CV%)		Accuracy (%)	Precision (CV%)
Diazepam	1	0.98 ± 0.04	97.52 ± 4.20	4.31	0.98 ± 0.10	98.16 ± 10.35	10.55
	3	2.91 ± 0.20	97.12 ± 6.69	6.89	2.87 ± 0.19	95.81 ± 6.31	6.58
	50	49.06 ± 1.88	98.12 ± 3.75	3.82	49.56 ± 1.36	99.12 ± 2.72	2.74
	250	245.29 ± 3.29	98.12 ± 1.32	1.34	243.58 ± 10.53	97.43 ± 4.21	4.32
	1000	1007.36 ± 15.81	100.74 ± 1.58	1.57	984.80 ± 40.51	98.48 ± 4.05	4.11
Nordazepam	1	1.03 ± 0.09	102.83 ± 8.85	8.60	1.00 ± 0.08	100.33 ± 7.74	7.71
	3	2.92 ± 0.19	97.21 ± 6.44	6.62	2.78 ± 0.30	92.76 ± 10.06	10.84
	50	52.35 ± 4.03	104.70 ± 8.07	7.71	49.82 ± 4.00	99.64 ± 8.00	8.03
	250	259.73 ± 13.95	103.89 ± 5.58	5.37	244.04 ± 19.65	97.62 ± 7.86	8.05
	1000	1060.60 ± 57.56	106.06 ± 5.76	5.43	992.10 ± 79.10	99.21 ± 7.91	7.97
Oxazepam	1	1.08 ± 0.05	107.52 ± 5.04	4.68	1.01 ± 0.13	101.43 ± 13.12	12.93
	3	2.72 ± 0.08	90.71 ± 2.83	3.12	2.77 ± 0.20	92.25 ± 6.83	7.40
	50	49.56 ± 1.38	99.13 ± 2.77	2.79	49.49 ± 0.99	98.97 ± 1.97	1.99
	250	244.74 ± 6.09	97.89 ± 2.44	2.49	243.64 ± 9.98	97.45 ± 3.99	4.10
	1000	1025.32 ± 14.78	102.53 ± 1.48	1.44	1002.18 ± 37.13	100.22 ± 3.71	3.71

Note: This table presents inter-day and intra-day precision and accuracy data for clear data classification and comparison. CV, coefficient of variation.

### 3.6 Matrix Effect, Extraction Recovery

Extraction recoveries and matrix effects of the analytes were determined at five QC concentrations (LLOQ, LQC, GMQC, MQC, and HQC). The average IS normalized matrix effect was 98%–102%. The average extraction recovery was 95%–101%, with the CV <6%. The CV of the IS normalized matrix factor at low and high concentration levels of DIA was 4.58% and 1.80%, those of NorD were 7.01% and 8.16%, and those of OXAZ were 3.57% and 1.22%, respectively, which were all <15%. Table 2 indicates that the matrix effect had a negligible impact on sample determination and provides details of the matrix effects and extraction recoveries.

**Table 2.** **Extraction recoveries, internal-standard-normalized matrix effects, and matrix effects of analytes in human serum samples (n = 9)**.

Analyte	Nominal Conc. (ng/mL)	Matrix effect		Recovery	
		Matrix effects (mean ± SD, %)	CV (%)	Extraction recovery (mean ± SD, %)	CV (%)
Diazepam	3	98.31 ± 0.045	4.58	101.91 ± 5.75	5.64
	1000	99.04 ± 0.018	1.80	95.27 ± 2.34	2.45
Nordazepam	3	102.49 ± 0.071	7.01	98.59 ± 1.21	1.23
	1000	100.40 ± 0.082	8.16	97.37 ± 1.67	1.71
Oxazepam	3	99.19 ± 0.035	3.57	100.29 ± 3.37	3.36
	1000	102.22 ± 0.013	1.22	97.65 ± 0.86	0.88

### 3.7 Stability

Table 3 provides an overview of the stability investigations on three substances in human serum under various storage regimes. The results of the QC samples (LQC and HQC) of the three drugs, which were placed at room temperature for 1 day, kept in the dark for 24 h after sample treatment and before injection, stored at –80 °C for 7 and 24 days, and frozen-thawed at –80 °C three times were stable, indicating that the investigated compounds did not degrade under the above conditions.

**Table 3.** **Stability study of the quality control samples (LQC and HQC) of the three target drugs (n = 3)**.

Storage Condition (ng/mL, mean ± SD)	Diazepam		Nordazepam		Oxazepam	
	3 ng/mL (mean ± SD)	1000 ng/mL (mean ± SD)	3 ng/mL (mean ± SD)	1000 ng/mL (mean ± SD)	3 ng/mL (mean ± SD)	1000 ng/mL (mean ± SD)
Serum at room temperature, 1 d	2.88 ± 0.04	1085.06 ± 18.32	3.16 ± 0.14	1042.30 ± 42.02	2.87 ± 0.09	1084.38 ± 7.91
Three freeze−thaw cycles, 3 cycles	3.27 ± 0.25	1121.35 ± 101.48	3.26 ± 0.21	1122.02 ± 101.58	3.01 ± 0.22	1120.18 ± 6.95
Prepared sample in autosampler at room temperature, 24 h	2.89 ± 0.15	1079.32 ± 12.86	2.98 ± 0.25	1071.77 ± 44.81	2.75 ± 0.09	1083.26 ± 4.41
Serum stored at −80 °C, 7 days	3.03 ± 0.06	1074.47 ± 36.90	2.87 ± 0.12	1125.93 ± 60.14	2.95 ± 0.02	1116.05 ± 21.77
Serum stored at −80 °C, 24 days	3.16 ± 0.04	914.17 ± 106.02	2.66 ± 0.18	892.1983 ± 133.40	2.73 ± 0.16	931.10 ± 124.82

LQC, low quality control; HQC, high quality control.

### 3.8 Clinical Application

**Fig. 3.**
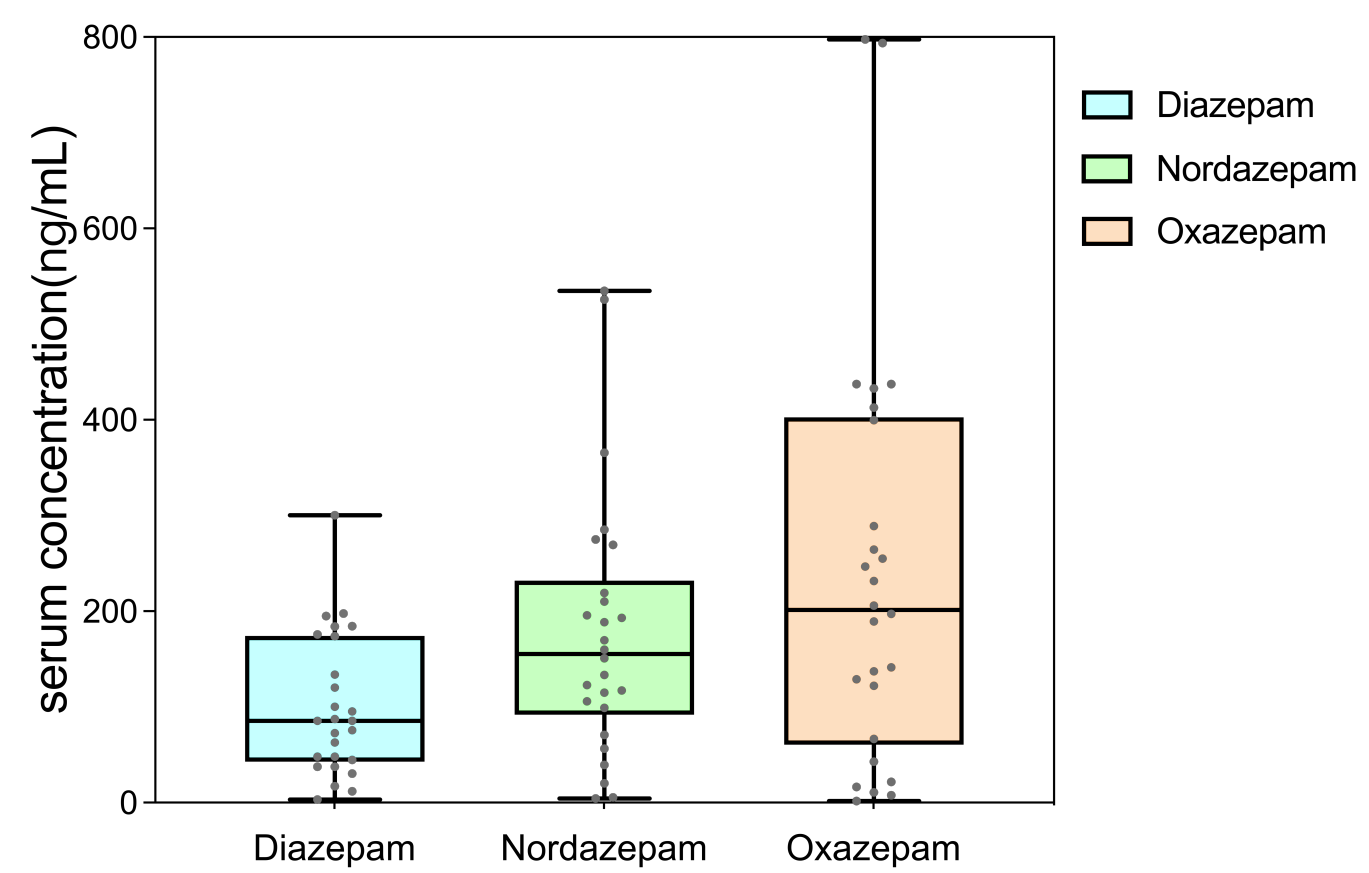
**Boxplot of serum concentrations of Diazepam (DIA), Nordazepam (NorD), and Oxazepam (OXAZ)**.

We performed TDM in 26 inpatients admitted to our hospital and diagnosed with alcohol dependence (24 males/2 females), with an average age of 40.7 ± 10.8 years (range 23–60 years) and average body mass index of 22.9 ± 3.6 kg/m^2^ (range 15.5–31.2 kg/m^2^). Patients were routinely treated with DIA alone (7.5–20 mg/day); DIA and OXAZ in combination (DIA 15–20 mg/day, OXAZ 45–105 mg/day); or OXAZ (15–90 mg/day) sequentially following DIA. The serum concentrations of DIA, NorD, and OXAZ in these patients were 100.3 ± 73.2 ng/mL (Q1–Q3 = 41.5–163.8 ng/mL), 178.2 ± 137.4 ng/mL (Q1–Q3 = 100.8–216.9 ng/mL), and 241.9 ± 216.7 ng/mL (Q1–Q3 = 54.8–344.2 ng/mL). The boxplot of the concentrations of DIA, NorD, and OXAZ is presented in Fig. 3. The DIA concentrations of serum samples from patients with alcohol dependence were lower than the generally reported range 200–1500 ng/mL [9, 28, 29]. According to the distribution range of sample concentrations, our method was sensitive enough and well-suited for the detection of serum concentrations in most patients with alcohol dependence treated with DIA and/or OXAZ. It should be noted that concentrations of DIA and NorD were still detectable in patients 2 or 3 days after discontinuation of DIA, suggesting that DIA and its metabolites still exert pharmacological activity after discontinuation.

## 4. Discussion

We used the isotopes of target drugs, namely DIA-D5, NorD-D5, and OXAZ-D5, as ISs. For NorD-D5, due to the influence of the sensitivity and response of the instrument and the purity of its deuterated IS, we needed a deuterated IS with higher purity to better meet the determination requirements of actual samples. The mass spectrometer had an acceptable response and good stability at 1 ng/mL. From the actual sample results, the standard curve concentration range of 1–1500 ng/mL met the needs of quantitative analysis and detection of DIA and its metabolites. The endogenous components in human serum did not obstruct the determination of the three target compounds and ISs, and DIA, NorD, OXAZ, and their ISs could be separated. The RSD of inter- and intra-assay precision and the coefficient of variation of matrix factor normalized by IS were both <15%. Serum samples showed good stability under different conditions.

Serum concentrations of DIA, NorD, and OXAZ could be simultaneously detected using the UPLC-MS/MS method in this study. Our approach offered good specificity, high sensitivity, accuracy, cost-effectiveness, eco-friendliness and good stability while requiring fewer serum samples and a total run time of 2.8 min per sample. In previous studies, SPE methods were used for sample preparation [14, 15, 16, 17, 25] in the development of a bioassay for quantitative assessment of DIA in human serum. However, SPE is complex and time-consuming for sample preparation [16, 17]. Compared with solid–liquid extraction (SLE) and LLE [19], protein precipitation with acetonitrile was simpler, analysis was shorter, and high-throughput quantitative analysis of serum samples was quicker. The acetonitrile protein precipitation method represents a green and efficient approach for serum sample preparation [26]. It reduces solvent consumption and time and achieves excellent extraction and separation efficiency, aligning with the principles of green analytical chemistry [26, 33, 34]. The dual focus of green analytical chemistry on analytical rigor and environmental responsibility highlights the key direction for future development, which will facilitate the creation of more sustainable analytical methods.

Our method was successfully applied to the routine TDM of DIA, NorD, and OXAZ in clinical samples from patients with alcohol dependence. Generally, the total concentration of the parent drug and active metabolites can be used for dose adjustment guided by TDM. The real-world TDM samples from patients with alcohol dependence included steady-state trough samples (n = 11) and samples from patients receiving sequential therapy (n = 15) who switched from DIA to OXAZ at their sampling time. Therefore, in this study, patients who received sequential OXAZ therapy after DIA were excluded from the subsequent analysis. For the steady-state trough samples, the concentrations of DIA and NorD were 158.38 ± 71.65 ng/mL and 246.92 ± 181.16 ng/mL, respectively. Compared with the pharmacokinetic data of DIA in healthy subjects reported in the literature [12, 35], the concentrations of DIA and NorD observed in our study were slightly higher. The total concentrations of DIA and NorD were 405.30 ± 231.47 ng/mL (range: 173.83–636.77 ng/mL), the concentration/dose ratio (C/D) of DIA was 12.45 ± 6.55 ng/mL/mg (range: 5.90–19.00 ng/mL/mg), the C/D of NorD was 20.56 ± 19.44 ng/mL/mg (range: 1.12–40.00 ng/mL/mg). The ratio of NorD/DIA was 1.41 ± 0.93 (range: 0.48–2.35), which was consistent with the literature-reported values [9]. According to the AGNP guidelines, the therapeutic concentration range of DIA plus metabolites, calculated based on steady-state trough concentrations, was 100–2500 ng/mL for patients with anxiety and sleep disorders. However, there was no consensus on the reference range of therapeutic concentration for patients with alcohol dependence. Several studies [9, 26, 28] reported the concentration range of DIA, which was consistent with our results, though slightly lower. Variations in dosing across different disease states may have contributed to the narrower concentration range observed in our study. The metabolite-to-parent compound ratio (MPR) is a direct indicator of metabolic enzyme activity at steady-state trough concentrations. MPR can identify abnormal metabolism caused by pharmacokinetic interactions or genetic variations; for example, a high MPR indicates enhanced enzyme activity. Additionally, changes in MPR can accurately reflect patient compliance issues. In this study, three patients (27%) had Nord/DIA ratios exceeding the expected MPR range (0.94–1.92) of the guideline, which may be attributed to enhanced CYP2C19 or CYP3A4 enzymatic activity or poor adherence. Thus, the disease status of patients with alcohol dependence, medication adherence, and changes in hepatic enzyme activity may influence DIA metabolism. In clinical practice, actual trough concentration samples for most psychotropic drugs should be collected in the morning before drug administration, 1 week after fixed-dose medication, typically 12–16 h after the last dose, i.e., at the end of the β-elimination phase. If the drug is taken within hours before blood collection, the concentration may reach several times the trough level (peak concentration) and exceed the reference range. Therefore, blood collection time directly affects the interpretation of TDM results. The current method provides a basis for subsequent studies of mechanisms of action, potential targets, dose selection, and investigation of therapeutic concentration ranges of DIA and OXAZ in the treatment of alcohol dependence.

## 5. Conclusions

We developed and validated a rapid, simple, and economic UPLC-MS/MS method for the quantification of DIA, NorD, and OXAZ in human serum. Serum samples were prepared by a one-step protein precipitation with acetonitrile. An isocratic elution was used, with a methanol–water system containing ammonium formate as the buffer to separate the analytes. The present method showed no matrix interference, satisfactory specificity and sensitivity, and appropriate recovery. The concentration range of 1–1500 ng/mL demonstrated acceptability in accuracy and precision. The method was well-suited for the determination of serum levels of DIA and its active metabolites in patients with alcohol dependence, and could be further applied to TDM and subsequent studies.

## Data Availability

The data that support the findings of this study are available from the corresponding author upon reasonable request.

## Abbreviations

UPLC-MS/MS, ultra-high performance liquid chromatography–tandem mass spectrometry; ESI, electrospray ionization; GABA, γ-aminobutyric acid; UV, ultraviolet; LLOQ, lower limit of quantitation; ULOQ, upper limit of quantification; SLE, solid–liquid extraction; LLE, liquid–liquid extraction; PP, precipitation of protein; SPE, solid phase extraction; QC, quality control; MRM, multiple reaction monitoring; RSD, relative standard deviation; MF, matrix factor; IS, internal standards; AGNP, Arbeitsgemeinschaft für Neuropsychopharmakologie und Pharmakopsychiatri; DIA, diazepam; NorD, nordazepam; OXAZ, oxazepam; CV, coefficient of variation; LQC, low quality control; GMQC, general medium quality control; MQC, medium quality control; HQC, high quality control; TDM, therapeutic drug monitoring.

## Supplementary Material

Supplementary material associated with this article can be found, in the online version, at https://doi.org/10.31083/AP38973.
