# Supplementary material for: UPLC-MS/MS Analytical Method for the Simultaneous Quantification of Diazepam, Nordazepam, and Oxazepam in Patients With Alcohol Dependence
Source: Alpha Psychiatry. 2026 Feb 26;27(1):38973. doi: 10.31083/AP38973 (PMC12957979; doi:10.31083/AP38973)
Supplement: Supplementary file 1 [file 2757-8038-27-1-38973-s1.zip › Supplementary Material.docx]

**Supplementary Table 1.** Comparison of sample preparation and analysis methods for diazepam (DIA), nordazepam (NorD), and oxazepam (OXAZ).

| **Author, Year [Ref.]** | **Matrix** | **Instrument** | **Extraction  Method** | **Extraction Solvent/Sorbent** | **Stationary phase** | **Mobile Phase** | **LLOQ (ng/mL)** | | | **Sample**  **Volume** | **Run Time (min)** | **Lineary Range** | **IS** | **Retention time (min)** |
| --- | --- | --- | --- | --- | --- | --- | --- | --- | --- | --- | --- | --- | --- | --- |
|  |  |  |  |  |  |  | DIA | NorD | OxaZ |  |  |  |  |  |
| Banaszkiewicz, 2020 [1] | blood | LC-MS /MS | LLE | Ethyl Acetate | Agilent Technologies InfinityLab Poroshell 120 EC-C18 column (3.0 mm × 100 mm, 2.7μm) + InfinityLab Poroshell 120 EC-C18 Fast Guard (3.0 mm × 5 mm, 2.7μm) | 0.05% formic acid in water (v/v, component A) and 0.05% formic acid in ACN (v/v, component B) | 1 | 1 | 1 | 0.5ml | 17 | DIA:1-200 ng/mL,  NorD:1-200 ng/mL,  OXAZ:1-200 ng/mL | DIA-D5 | OXAZ: 6.46; NorD: 6.93;  DIA: 7.92 |
| Wang, 2020 [2] | blood | LC-MS/MS | SPE | C18 | Agilent HPLC C18 (3.0 × 50 mm, 2.7 μm) | solvent A (acetoni- trile) and solvent B (ultrapure water) | 0.1 | 0.1 | 0.5 | 1mL | 17 | DIA: 0.1-100 ng/mL, NorD: 0.1-100 ng/mL, OXAZ: 0.5-100 ng/mL | NorD-d5 | - |
| Barone, 2023 [3] | serum or blood | UPLC-MS/MS | PP | Acetonitrile | Acquity UPLC® HSS C18 column (2.1×150mm, 1.8 µm; Waters) | mobile phase A composed of aqueous solution 5 mM ammonium formate and mobile phase B composed of acetonitrile, both spiked with 0.1% formic acid | 64 | 58 | 58.5 | 100 μL | 15 | DIA: 64.00-1733.0 ng/mL,  NorD: 58.00-1653.0 ng/mL,  OXAZ: 58.50-1808.0 ng/mL | NorD -D5 | OXAZ: 8.02; NorD: 9.08;  DIA: 10.56 |
| Agarwal,2013 [4] | blood | LC-MS/MS | SPE | methanol | - | - | 0.1 | - | - | 50 μL | - | DIA: 0.1-100 ng/mL | DIA -d5 | - |
| Lee, 2017 [5] | blood | UFLC-MS/MS | on-line column-switching UFLC-MS/MS | 13.3 mM ammonium acetate/acetonitrile (33:67, v/v) | Oasis HLB cartridge (20 mm × 2.1 mm, 25 μm,) and a SUMIPAX ODS D-Swifter column (30 mm × 3.0 mm, 2 μm,) | 10 mM ammonium acetate (pH 6.76) (solvent A) and acetonitrile (solvent B) | 0.05 | - | - | 100 μL | 7 | DIA:0.05-2 ng/mL | OXAZ-d5 | DIA: 3.65 |
| Wang, 2013 [6] | blood | LC–MS/ MS | SPE | Waters Oasis HLB 96-well μElution SPE plate | Eclipse XDB C18 column | solvent A (2 mM ammonium formate and 0.1% formic acid in water) and solvent B (2 mM ammonium formate and 0.1% formic acid in acetonitrile) | 0.1 | 0.1 | 0.2 | 200 μL | 23 | DIA: 0.1-200 ng/mL, NorD: 0.1-200 ng/mL, OXAZ: 0.2-400 ng/mL | DIA- d5, OXAZ-d5, and Estazepam-d5 | DIA: 13.85; NorD: 11.79; OXAZ: 10.43 |
| Jiang, 2016 [7] | oral fluid | LC– MS/MS | online SPE | HySphere Resin GP cartridge | Waters XterraC18 column | solvent A (2 mM ammonium formate and 0.1% formic acid in water) and solvent B (2 mM ammonium formate and 0.1% formic acid in acetonitrile | 0.1 | 0.1 | 0.25 | 200 μL | 21 | DIA: 0.1-100 ng/mL,  NorD: 0.1-100 ng/mL,  OXAZ:0.25-250 ng/mL | DIA-d5, NorD -d5, and OXAZ-d5 | DIA: 9.1; NorD: 8.02; OXAZ: 7.41 |
| De Boeck, 2017 [8] | whole blood | LC-MS/MS | DLLME | ionic liquids (ILs), BMIm PF6 | a Kinetex® Biphenyl LC Column (100 mm x 2.1 mm, 2.6 μm particle size) | Aqueous buffer pH 8.0 and methanol | 50 | 10 | 50 | 1mL | 14 | DIA: 50-2000 ng/mL,  NorD:10-1000 ng/mL, OXAZ:50-2000 ng/mL | DIA-d5, NorD -d5, and OXAZ-d5 | DIA: 8.8;  NorD: 7.9; OXAZ: 7.2 |
| Kim, 2017 [9] | plasma | HPLC- MS/MS | LLE | ethyl acetate:n-hexane (80:20, v/v), | Phenomenex Cadenza CD-C18 column (150 × 3.0 mm, 3 µm) | 10 mM ammonium acetate in water:methanol [5:95, v/v] | 0.5 | - | - | 50 μL | 3 | DIA: 0.5-300 ng/mL | Voriconazole | DIA: 2.32 |
| Verplaetse, 2012 [10] | urine and blood | UFLC -MS/MS | SPE | 2 ml methanol and 2 ml 0.1 M phosphate buffer pH 6 | Acquity C18 column (2.1 mm × 50 mm, 1.7 μm) | 10 mM ammonium bicar- bonate in water set at pH 9.0 (solvent A) and methanol (solvent B) | 2 | 2 | 2 | 0.5ml | 13 | DIA: 2-500 ng/mL,  NorD: 2-500 ng/mL,  OXAZ: 2-500 ng/mL | N-methylclonazepam | DIA: 5.8;  NorD: 5.6;  OXAZ: 4.8; |
| Kristoffersen, 2018 [11] | whole blood | UPLC-MS/MS | SLE | ethyl acetate + heptane (80 + 20, v/v) | Acquity UPLC BEH C18 column (50 × 2.1 mm, 1.7 μm) with an Acquity UPLC BEH C18 pre-column (2.1 × 5 mm, 1.7 μm particles) | 5 mM pH 10.2 ammonium formate (A)and methanol(B) | 30 | 30 | 90 | 100μl | 4.5 | DIA: 200-10000 ng/mL, NorD: 200-10000 ng/mL, OXAZ: 600-15000 ng/mL | 13C6- DIA, 13C6- OXAZ, and 13C6- NorD | DIA: 2.54;  OXAZ:2.34;  Nord: 2.49 |
| Montenarh, 2014 [12] | Whole blood, plasma, and serum | LC-MS/MS | LLE | diethyl ether-ethyl acetate (50/50) | Waters SunFire C18 column (2.1 × 150 mm, 3.5 μm) | 10 mM aqueous ammonium formate plus 0.1 % formic acid pH 3.4 (eluent A) and acetonitrile plus 0.1 % formic acid (eluent B). | 1 | 0.1 | 0.2 | 500μl | 30 | DIA:100-3200 mg/mL, NorD: 100-3200 mg/mL, OXAZ: 200-3200 mg/mL | Trimipramine-d3 | - |
| Marin, 2012 [13] | serum | UPLC- MS/MS | LLE | dichloromethane–ether–hexane (30:50:20 containing 0.5% isoamyl alcohol) | An Acquity UPLC BEH C18 column (2.1 × 100 mm, 1.7mm) | 0.1% formic acid in water (A) and 0.1% formic acid in methanol (B) | - | - | - | 0.3ml | 7.5 | DIA: 1-1000 ng/mL,  NorD: 1-1000 ng/mL, OXAZ: 1-1000 ng/mL | DIA-d5, OXAZ-d5, and NorD -d5 | - |
| Rust, 2012 [14] | human hair | LC-MS/MS | LLE | methanolic and methanolic/aqueous | Phenomenex Kinetex, 2.6 mm, 50/2.1 | 5 mM ammonium formate buffer adjusted to pH 3.5 with formic acid (eluent A) and methanol containing ammonium formate (eluent B) | 10 | 10 | 10 | 30mg | - | DIA; 10-1600 pg/mg, NorD: 10-1600 pg/mg, OXAZ: 10-1600 pg/mg | DIA-d5, OXAZ-d5 | - |
| Bjørk, 2013 [15] | whole blood | UPLC-MS/MS | SPE | Strata X-C plates(MeOH  and water) | Acquity UPLC CSH C18 column(100 mm × 2.1 mm, 1.7 μm) | solvents A (0.1 % aqueous ammonia (25 %) and B (0.1 % aqueous ammonia (25 %) in methanol) | - | - |  | 200μl | 6.5 | DIA: 0.002-1 mg/kg,  NorD: 0.002-1 mg/kg | DIA-d5, OXAZ-d5, and NorD -d5 | DIA: 4.00；NorD: 3.84; OXAZ: 3.17 |
| Tok, 2025 [16] | Plasma | HPLC-UV | PP | 4% TCA: Acetonitrile (20:80 v/v) | Kinetex F5 column (150 ×4.6 mm, 5 µm) | acetonitrile and phosphate buffer in a ratio of 30:70 (v/v) | 200.0 | - | - | 100μl | 7 | DIA: 200-5000 ng/mL | Clozapine | DIA: 6.02；NorD: 4.12 |
| Qandeel, 2012 [17] | tablet | ^1^H-qNMR | - | 0.3 M sodium dodecyl sulfate prepared in deuterated water | - | - | - | - | - | - | - | DIA: 250-15000 ng/mL | Phloroglucinol | - |
| **This work** | **serum** | **UPLC-MS/MS** | **PP** | **Acetonitrile** | **Agilent XDB-C18 (50 mm×4.6 mm, 1.8 μm)** | **75% methanol (25/75, V/V, water/methanol) containing 5 mM ammonium formate** | **1** | **1** | **1** | **100μl** | **5** | **DIA: 1-1500 ng/mL, NorD: 1-1500 ng/mL, OXAZ: 1-1500 ng/mL** | **DIA-d5, OXAZ-d5, and NorD -d5** | **DIA: 2.2;**  **NorD: 2.0; OXAZ: 1.6** |

- **EA:** Ethyl Acetate, **ACN:** Acetonitrile, **LLE:** Liquid-Liquid Extraction, **PP:** Protein precipitation, **SPE:** Solid Phase Extraction, **BMIm PF6**:1-butyl-3-methylimidazolium hexafluorophosphate, **DLLME**: dispersive liquid liquid microextractions, **SLE**: supported liquid extraction

References and Notes

[1] Banaszkiewicz L, Woźniak MK, Kata M, et al. Rapid and simple multi-analyte LC-MS/MS method for the determination of benzodiazepines and Z-hypnotic drugs in blood samples: Development, validation and application based on three years of toxicological analyses. Journal of Pharmaceutical and Biomedical Analysis. 2020, 191:113569. https://doi.org/10.1016/j.jpba.2020.113569.

[2] Wang LL, Ren XX, He Y, et al. Study on the Pharmacokinetics of Diazepam and Its Metabolites in Blood of Chinese People. European Journal of Drug Metabolism and Pharmacokinetics. 2020, 45(4):477-485. https://doi.org/10.1007/s13318-020-00614-8.

[3] Barone R, Giorgetti A, Cardella R, et al. Development and validation of a fast UPLC-MS/MS screening method for the detection of 68 psychoactive drugs and metabolites in whole blood and application to post-mortem cases. Journal of Pharmaceutical and Biomedical Analysis. 2023, 228:115315. https://doi.org/10.1016/j.jpba.2023.115315.

[4] Agarwal SK, Kriel RL, Brundage RC, et al. A pilot study assessing the bioavailability and pharmacokinetics of diazepam after intranasal and intravenous administration in healthy volunteers. Epilepsy Research. 2013, 105(3):362-7. https://doi.org/10.1016/j.eplepsyres.2013.02.018.

[5] Lee XP, Shouji Y, Kumazawa T, et al. Rapid and highly sensitive analysis of benzodiazepines and tandospirone in human plasma by automated on-line column-switching UFLC-MS/MS. Legal Medicine (Tokyo). 2017, 24:36-55. https://doi.org/10.1016/j.legalmed.2016.11.005.

[6] Wang R, Wang X, Liang C, et al. Direct determination of diazepam and its glucuronide metabolites in human whole blood by muElution solid-phase extraction and liquid chromatography-tandem mass spectrometry. Forensic Science International. 2013, 233(1-3):304-11. https://doi.org/10.1016/j.forsciint.2013.10.004.

[7] Jiang F, Rao Y, Wang R, et al. Sensitive, automatic method for the determination of diazepam and its five metabolites in human oral fluid by online solid-phase extraction and liquid chromatography with tandem mass spectrometry. Journal of Separation Science. 2016, 39(10):1873-83. https://doi.org/ 10.1002/jssc.201600107.

[8] De Boeck M, Missotten S, Dehaen W, et al. Development and validation of a fast ionic liquid-based dispersive liquid-liquid microextraction procedure combined with LC-MS/MS analysis for the quantification of benzodiazepines and benzodiazepine-like hypnotics in whole blood. Forensic Science International. 2017, 274:44-54. https://doi.org/10.1016/j.forsciint.2016.12.026.

[9] Kim DH, Cho JY, Chae SI, et al. Development of a simple and sensitive HPLC-MS/MS method for determination of diazepam in human plasma and its application to a bioequivalence study. Translational and Clinical Pharmacology. 2017, 25(4):173-178. https://doi.org/10.12793/tcp.2017.25.4.173.

[10] Verplaetse R, Cuypers E,Tytgat J. The evaluation of the applicability of a high pH mobile phase in ultrahigh performance liquid chromatography tandem mass spectrometry analysis of benzodiazepines and benzodiazepine-like hypnotics in urine and blood. Journal of Chromatography A. 2012, 1249:147-54. https://doi.org/10.1016/j.chroma.2012.06.023.

[11] Kristoffersen L, Langødegård M, Gaare KI, et al. Determination of 12 commonly found compounds in DUID cases in whole blood using fully automated supported liquid extraction and UHPLC-MS/MS. Journal of Chromatography B: Analytical Technologies in the Biomedical and Life Sciences. 2018, 1093-1094:8-23. https://doi.org/10.1016/j.jchromb.2018.06.050.

[12] Montenarh D, Hopf M, Maurer HH, et al. Detection and quantification of benzodiazepines and Z-drugs in human whole blood, plasma, and serum samples as part of a comprehensive multi-analyte LC-MS/MS approach. Analytical and Bioanalytical Chemistry. 2014, 406(3):803-18. https://doi.org/10.1007/s00216-013-7513-x.

[13] Marin SJ, Roberts M, Wood M, et al. Sensitive UPLC-MS-MS assay for 21 benzodiazepine drugs and metabolites, zolpidem and zopiclone in serum or plasma. Journal of Analytical Toxicology. 2012, 36(7):472-6. https://doi.org/10.1093/jat/bks059.

[14] Rust KY, Baumgartner MR, Meggiolaro N, et al. Detection and validated quantification of 21 benzodiazepines and 3 "z-drugs" in human hair by LC-MS/MS. Forensic Science International. 2012, 215(1-3):64-72. https://doi.org/10.1016/j.forsciint.2011.07.052.

[15] Bjørk MK, Simonsen KW, Andersen DW, et al. Quantification of 31 illicit and medicinal drugs and metabolites in whole blood by fully automated solid-phase extraction and ultra-performance liquid chromatography-tandem mass spectrometry. Analytical and Bioanalytical Chemistry. 2013, 405(8):2607-17. https://doi.org/10.1007/s00216-012-6670-7.

[16] Tok KC, Ozkan-Kotiloglu S, Bozmaoglu CH, et al. Development of a sample preparation and analysis method for therapeutic monitoring of diazepam and major metabolite in alcohol withdrawal syndrome treatment. Journal of Pharmaceutical and Biomedical Analysis. 2025, 260:116805. https://doi.org/10.1016/j.jpba.2025.116805

[17] Qandeel NA, El-Masry AA, El-Shaheny R, et al. Utility and greenness appraisal of nuclear magnetic resonance. for sustainable simultaneous determination of three 1,4-benzodiazepines and their main impurity 2-amino-5-chlorobenzophenone. Scientific Reports. 2023, 13(1):21121. https://doi.org/10.1038/s41598-023-48416-7.
